# Urine and stone analysis for the investigation of the renal stone former: a consensus conference

**DOI:** 10.1007/s00240-020-01217-3

**Published:** 2020-10-13

**Authors:** James C. Williams, Giovanni Gambaro, Allen Rodgers, John Asplin, Olivier Bonny, Antonia Costa-Bauzá, Pietro Manuel Ferraro, Giovanni Fogazzi, Daniel G. Fuster, David S. Goldfarb, Félix Grases, Ita P. Heilberg, Dik Kok, Emmanuel Letavernier, Giuseppe Lippi, Martino Marangella, Antonio Nouvenne, Michele Petrarulo, Roswitha Siener, Hans-Göran Tiselius, Olivier Traxer, Alberto Trinchieri, Emanuele Croppi, William G. Robertson

**Affiliations:** 1 Department of Anatomy, Cell Biology & Physiology, Indiana University School of Medicine, Indianapolis, IN 46260 USA; 2 Division of Nephrology and Dialysis, Department of Medicine, University of Verona, Verona, Italy; 3 Department of Chemistry, University of Cape Town, Cape Town, South Africa; 4 Litholink, Laboratory Corporation of America Holdings, Itasca, IL USA; 5 Service of Nephrology, Department of Medicine, Lausanne University Hospital and Department of Biomedical Sciences, University of Lausanne, Lausanne, Switzerland; 6 IUNICS-Idisba, University of Balearic Islands, Palma de Mallorca, Spain; 7 UOC Nefrologia, Fondazione Policlinico Universitario A. Gemelli IRCCS, and Università Cattolica del Sacro Cuore, Rome, Italy; 8 Policlinico di Milano, Milan, Italy; 9 Inselspital, Bern University Hospital, University of Bern, Bern, Switzerland; 10 NYU Langone Health, NYU Grossman School of Medicine and the New York Harbor VA Healthcare System, New York, NY USA; 11 Universidade Federal de São Paulo, São Paulo, SP Brazil; 12 SAELO, Oegstgeest, The Netherlands; 13 Service des Explorations Fonctionnelles Multidisciplinaires, Hôpital Tenon, Paris, France; 14 Laboratory of Clinical Chemistry and Hematology, University Hospital of Verona, Verona, Italy; 15 Fondazione Scientifica Mauriziana ONLUS, Turin, Italy; 16 Internal Medicine and Subacute Long Term Unit, Azienda Ospedaliero-Universitaria di Parma, Parma, Italy; 17 Ospedale Ordine Mauriziano di Torino, Turin, Italy; 18 University Stone Center, Department of Urology, University Hospital Bonn, Bonn, Germany; 19 Department of Clinical Science, Intervention and Technology (CLINTEC), Karolinska Institutet, Stockholm, Sweden; 20 Service d’Urologie, Hôpital Tenon, Paris, France; 21 School of Urology, University of Milan, Milan, Italy; 22 USL Toscana Centro, Florence, Italy; 23 Nuffield Department of Surgical Sciences, University of Oxford, Oxford, UK

**Keywords:** Nephrolithiasis, Urine analysis, Stone analysis, Crystalluria

## Abstract

The Consensus Group deliberated on a number of questions concerning urine and stone analysis over a period of months, and then met to develop consensus. The Group concluded that analyses of urine and stones should be routine in the diagnosis and treatment of urinary stone diseases. At present, the 24-h urine is the most useful type of urine collection, and accepted methods for analysis are described. Patient education is also important for obtaining a proper urine sample. Graphical methods for reporting urine analysis results can be helpful both for the physician and for educating the patient as to proper dietary changes that could be beneficial. Proper analysis of stones is also essential for diagnosis and management of patients. The Consensus Group also agreed that research has shown that evaluation of urinary crystals could be very valuable, but the Group also recognizes that existing methods for assessment of crystalluria do not allow this to be part of stone treatment in many places.

**Electronic supplementary material:**

The online version of this article (10.1007/s00240-020-01217-3) contains supplementary material, which is available to authorized users.

## Introduction

The use of urine analysis as a guide to the diagnosis and treatment of kidney stones is recommended for at least some stone formers in all of the published international guidelines [1–4] (see Supplemental Table 1), but data suggest it is not generally utilized as widely as has been recommended. For example, a recent study of a large cohort within the United States (US) Veterans Affairs Health Care System found that fewer than 1-in-6 stone forming patients had undergone 24-h urine testing that would have been relevant to managing their urinary stone disease [5]. A possible interpretation of this low utilization of urine data is that physicians in the US are unconvinced that urine testing is valuable and cost-effective [6–8].

In the UK, most health authorities have abandoned the routine biochemical screening of stone patients to save money in favour of managing kidney stone patients solely through the urological removal or disintegration of their stones. This approach, in itself, does not “cure” the patients’ underlying risk of forming further stones. Furthermore, in general, no preventative treatment is instituted and so most patients return with further stones at a later date. It has been shown that this strategy actually costs more than would be the case if proper biochemical screening were to be instituted thereby resulting in a reduction in stone recurrence [9].

In contrast, within specialist stone centres for the treatment of stone patients in Europe, the use of urine analysis for patient management is relatively uniform, with a recent survey showing that 96% of stone centres perform 24-h urine analyses as part of both initial and follow-up visits for patients [10]. However, these specialist stone centres did not agree on the best methods for collection and analysis of a 24-h urine specimen, and only 3 of the 24 stone centres that were surveyed commented that they used any calculation of the supersaturation levels of urinary salts and acids to assess their patients’ risks of forming further stones. Thus, even in health centres that specialize in the management of stone patients, there exists some uncertainty about how urine analyses should be performed and how the data should be interpreted.

A lack of uniformity also exists regarding methods of stone analysis, which is essential to the interpretation of urine analyses [11]. In the recent survey of European stone centres, 21% of the centres reported using only wet chemical, rather than spectroscopic methods for stone analysis [10], even though the use of chemical analysis has repeatedly been shown to result in serious errors that can lead to incorrect clinical conclusions [12, 13]. Finally, the identification of crystals in urine has not gained much traction as an aid to diagnosing and managing the stone forming patient, even though some researchers suggest that the disappearance of crystalluria is the best evidence that clinical management has been effective in reducing the risk of stone recurrence [14, 15].

The purpose of the convocation of the 4^th^ International Meeting of the Menarini Foundation on Nephrolithiasis was to address these issues and to seek the consensus of an international Group of experts who specialize in various aspects of the use of urine, stone, and crystalluria analyses for treating stone patients. The Group worked to develop consensus on general topics (such as the value of urine analysis for treating stone forming patients) and on specific issues (such as how a specimen should be collected and analysed). The Group also sought to identify areas of study that would be especially fruitful for improving the scientific grounding of these practices.

### Brief of the Consensus Group

The brief which was assigned to the Group by the Meeting’s organizers was: (1) to assess the current evidence for the use of urine analysis in the treatment of nephrolithiasis and to recommend best practice thereof; (2) to specify practical recommendations for the optimum collection of urine specimens and what variables should be measured in the specimens; (3) to identify the most suitable methods for measuring urine properties and constituents, what can be measured at home, what supersaturation and risk indices should be calculated, and how the results should be communicated to the referring physician and to the patient, and (4) to recommend optimum methods for the analysis of stones and crystals passed in urine.

The Group initially performed its work by means of electronic communication over a period of 5 months prior to Meeting in June 2019 in Verona, where it sought to reach a consensus. This document provides a summary of the conclusions of the Group.

### References used

This document does not provide an exhaustive listing of all relevant research papers in the field. Instead, the works cited here have been selected as being the most appropriate for achieving consensus. All provide useful entry points into each subject area. However, an effort was made to ensure that no significant paper was ignored in the deliberations of the Consensus Group. This effort included solicitation of important papers from each of the members of the Group, followed by an appropriately extensive subject search using PubMed. Some key papers were also searched forward to look for citing articles using Web of Science (Clarivate).

## Q1. What is the value of collecting urine samples from patients with kidney stones?

The process of crystallization in urine is complex, but urine analysis allows identification of the potential key chemical factors which are thought to lead to the precipitation of minerals that form stones. Armed with such information, physicians can institute appropriate interventions to alter these factors with a view to reducing the risk of further stone formation.

Treatment regimens such as specific dietary recommendations (for example, to manage hypercalciuria and mild hyperoxaluria [16]), or administration of medications such as citrate (to increase pH and citrate in urine), thiazides (to decrease hypercalciuria), or allopurinol (to decrease hyperuricosuria) are generally not implemented without prior urine analysis to identify the underlying pathological conditions concerned. Moreover, most physicians who prescribe such medications want to follow up their patients with repeat urine analysis to ensure that the patient is complying with treatment and that the medication is having the desired effect. Urine analysis is, therefore, crucial for directing the clinician towards suitable treatment and for following up the patient over time.

Although it might be possible for a physician to prescribe medications such as citrate, thiazides, or allopurinol in an empiric manner, and without urine analysis results [7], this is not common practice. A recent study of 130,489 stone patients in the US veterans system showed that only a minority of patients (13%) underwent a 24-h urine workup, but that this minority was more likely to be prescribed citrate, thiazide, or allopurinol than were the stone formers who were not requested to collect a 24-h urine [17]. Importantly, their data also showed that prescription of these medications followed logically from the results of the patients' 24-h urines. Thus, physicians treating these stone patients were more likely to prescribe medication when the urine analysis data pointed to a specific need in the patient (e.g., to reduce hypercalciuria). Our consensus is that this is a rational approach, and that most physicians would not want to commit a patient to a medication without prior evidence from urine analysis. Indeed, a recent paper has shown that a decline in supersaturation values for calcium oxalate (CaOx) and in 24-h urine excretion of citrate, potassium and magnesium with treatment were associated with longer periods without recurrence [18]. The Consensus Group recognized that more studies, such as this one, would be helpful in establishing the value of therapy driven by urine analysis in stone patients.

Urine analysis also forms part of the work-up of a stone-forming patient for the identification of diseases predisposing to or associated with nephrolithiasis. These conditions include primary hyperparathyroidism, primary hyperoxaluria, enteric hyperoxaluria, cystinuria, and distal renal tubular acidosis. The percentage of stone formers who have one of these conditions is not large, but recognizing these diseases is essential for proper treatment, and urine analysis is certainly part of that process.

The assessment of a patient’s diet to identify potential predisposing factors is another extremely important benefit which clinicians can derive from analysis of urine. This aspect is discussed in several sections which appear later in this document.

It was recognized by the Consensus Group that in most countries, detailed analysis of urine samples from stone formers tends to be limited to specialist laboratories and practices. The Group agreed that transfer of basic skills to general practitioners could benefit huge numbers of patients worldwide.

### Consensus on Q1

The consensus in the Group was that analysis of urine samples is essential for the meaningful management of stone formers. The Group recommends that urine analysis needs to be performed in conjunction with metabolic studies, stone analysis and dietary assessment.

### Q2. What type of urine collection is best for assessing a patient's risk of forming stones: a 24-h urine, or some other type of collection?

Numerous reports in the literature on patients with urolithiasis refer to results of analysis of 24-h urine composition. An overwhelming number have shown that 24-h urinary risk factors for stone-formation are more frequently abnormal in stone forming patients than in normal controls [7, 19–21]. Reports also show that 24-h abnormalities provide useful information to help understand the underlying cause of stones in a given patient as well as for targeting changes in urine composition that should reduce his/her risk of stone recurrence. Both the American Urological Association (AUA) and the European Association of Urology (EAU) in their guidelines recommend 24-h urine samples as the standard procedure for evaluation at least for calcium stone formers [1, 2]. In general, 24-h urine supersaturation levels may be used to predict the likelihood that a person is a stone former [22, 23].

Yet, as mentioned in the Introduction, remarkably few stone patients in the US are evaluated using 24-h urine data. A recent study of a very large cohort showed that patients who did not complete a 24-h urine study (87% of 130,489 patients, all of whom were within a system that would pay for their 24-h urine testing) were less likely to receive medications that are thought to reduce the risk for stone recurrence such as thiazides, alkali, citrate or allopurinol. Those who did collect a 24-h urine were prescribed medication that was clearly linked to the results of their 24-h urine analyses [17]. This points to the practical value of 24-h urine testing in providing the physician with data to prompt and guide the prescription of appropriate dietary advice or medication.

Most of the participating experts expressed their belief that 24-h urine analysis is essential for diagnosing and following up patients with calcium-containing stones, for the appropriate prescription of medication to reduce stone recurrence, for advising patients on dietary adjustments, and for verifying (and encouraging) patient compliance. Even in those few cases in which spot urine could suffice for the diagnosis (i.e., cystinuria or primary hyperoxaluria) 24-h urine collections are essential for monitoring not only the effects of treatment on urine composition, but also on compliance to the recommendation to increase water intake.

However, the efficacy of monitoring 24-h urine analysis during follow up with concomitant modification of dietary advice and drug treatment culminating in a decrease in stone recurrence is not established. Ferraro et al. [18] have investigated this issue using a post-hoc analysis of the Borghi trial [24]. Due to the experimental design only very short-term variations (1 week) were considered. A prospective, randomized trial has been conducted to compare recurrence in patients whose dietary recommendations were made with and without tests that included 24-h urine analysis [25], and the results of this trial support the value of 24-h urine testing in reducing stone recurrence. However, more long-term, randomized control trials involving large numbers of stone patients are needed to establish the actual value of 24-h urine testing in preventing stone recurrence.

The Group agreed that spot urines (including morning fasting urines) or other urine collections over part of a day can be valuable, too, but there are too few studies to identify best practice for urine analysis outside of the standard 24-h collection. In view of the well-known diurnal variation of urine composition in calcium stone forming patients [26–29], it seems certain that analysis of urine at specific times of day could be even more informative than the comprehensive 24-h urine. This is important, because in some patients (such as children) obtaining a 24-h urine specimen is impractical. A recent paper has suggested that an afternoon collection of urine in children may substitute in some ways for a 24-h urine specimen [30]. In adults, there is evidence of variation among individuals in their diurnal patterns of urine composition [29], which complicates this approach. However, a recent proof-of-concept study on healthy volunteers has suggested that it might be rational to investigate specific timed urine collections according to the stone type (e.g., 8 pm to 8 am for calcium oxalate, 2–4 pm for uric acid, or any 2-h timed urine collection during daytime for calcium phosphate [31]. More studies in this direction are likely to be helpful.

### Consensus on Q2

The Consensus Group agreed that 24-h urine analysis is essential for diagnosing and following stone patients and should be the main modality utilized in most practices. However, the Group recognizes the significant value of other urine collection modalities and encourages further research in this area.

## Q3. How many urine collections should be made from a given patient and how frequently should follow-up urine samples be collected to monitor the patient's progress?

Studies have shown that a single urine collection often does not provide a complete picture of the abnormalities in a stone patient, and that collection of two urine samples for analysis is generally better [32–34]. One practical aspect of having at least two analyses is that variations in dietary factors can sometimes be easily identified (such as when oxalate excretion varies dramatically between two days) and identification of relevant conditions such as hypercalciuria can be more accurately established [32].

It is also recognised that the diagnostic accuracy of 24 h-urine testing not only increases with the number of urines collected, but also with time passing before urine collections after a stone event, most likely due to a vanishing ‘stone clinic effect’ [35].

Patient education is also important for proper urine collection, both to motivate the patient to complete the collections properly and to ensure that the collections represent the patient’s normal lifestyle practices. For the two 24-h urine samples at initial evaluation, it is wise to coach the patient that one collection should be done on a day at work and one performed on a non-working day. Left to their own choice, a patient is likely to want to collect both specimens at home, yet most people spend more days at work than at home and the environmental and nutritional differences may be critical in determining their stone risk. Most importantly, 24-h urine collections should only be carried out when the patients are consuming their free, everyday diets—never as in-patients, since the foods and drinks served in hospital may be very different from those normally consumed by the patients at home.

The frequency of follow-up analyses should be determined by the kind of treatment that has been prescribed, but in most cases an initial follow-up at 3–6 months is warranted [36, 37]. If no changes are made in treatment, then annual checks thereafter are probably sufficient for tracking.

### Consensus on Q3

The Consensus Group recommends two 24-h urine analyses for initial evaluation of stone patients. One collection should be made on a workday and the other should be made during a non-workday. It is important that the patients should collect their 24-h urines when they are consuming their free, everyday diets. Follow-up at 3–6 months is recommended. The Consensus Group emphasizes that patient education on how and when to collect their 24-h urine samples is important.

## Q4. Is urine composition a reliable indicator of the patient's diet?

It is well known that dietary habits affect the risk of kidney stone formation. There are a number of ways to assess a patient’s diet: (1) diet diary; (2) 24-h diet recall; (3) food frequency assessment; and (4) 24-h urine chemistries. These approaches have strengths and weaknesses, but the use of 24-h urine composition in conjunction with one of the diet history approaches may provide the most comprehensive way to assess the role of diet and its effect on stone risk.

Urine data provide an objective measure that does not depend on patient recall (with its possible bias), and the data relevant to diet can be interpreted quickly and easily. The dietary data derived from the 24-h urine analysis also allow assessment of the possible metabolic interaction between certain dietary factors that may have an effect on urinary risk factors for stones; for example, if the patient has a high urine calcium it is important to know if markers of sodium and protein intake are high for the same day [38, 39]. Multiple 24-h urine collections also provide an estimate of dietary variance [34, 40].

Dietary sodium intake is notoriously difficult to estimate by history or food frequency questionnaire. Salt added during food preparation is often unquantified as is salt added at mealtime. Urine sodium excretion is considered the most reliable method to assess sodium intake [41]. Confounding factors include excessive sweating, diarrheal diseases, and sodium retention (such as during menstruation), all of which can lead to discrepancy between sodium intake and urine sodium excretion, but in general urinary sodium excretion is a sufficiently accurate measure of dietary sodium intake to be a useful clinical tool [42, 43].

Urine potassium can be used as a marker of dietary potassium intake. Approximately 85 to 90% of diet potassium is absorbed assuming normal gut function [42]. In steady state, urine potassium excretion will approximate intestinal potassium absorption. Because potassium is predominantly an intracellular cation there is considerable capacity to accept the dietary load, leading to some offset in time from ingestion to excretion of a potassium load. However, there is still good agreement of intake to excretion [43]. Urine potassium excretion can be particularly useful in monitoring adherence to prescribed potassium alkali.

Urine urea can be used as a marker of total protein intake [42], while sulphate is as a marker of animal protein intake, as sulphur amino acids are oxidized to sulfuric acids, which is excreted as sulphate [42, 44]. These two markers are highly correlated, and either can be used to monitor protein intake. As sulphate is probably not measured routinely in all stone clinics, it should be mentioned that uric acid is also a reliable marker of animal (non-dairy) protein, because—in comparison with other urinary protein markers such as phosphate and sulphate—only uric acid increases significantly on certain acid-rich diets [45].

Despite these strengths, there are weaknesses in using 24-h urine data alone to assess diet. This method will yield data only for a single day’s diet and will not provide a measure of fat or carbohydrate intake. Moreover, the urine data alone will not allow an assessment of calcium or oxalate intake. A further limitation of using the 24-h collection for dietary assessment is that it is possible that some patients may temporarily increase their compliance with their dietary advice in an attempt to “improve” the composition of their urine collection, which they can accomplish easily for a single day. This applies particularly to the intake of fluid and its effect on urine volume. It is recognised that patients often try to “please” their doctor by drinking more on the day before and during the day of their urine collection. The Internet may also play a significant role in directing patients (rightly or wrongly) as to what to eat and drink to self-treat their stone problem.

In addition to providing a primary therapeutic endpoint for solutes, passing a sufficiently large urine volume is critical for reducing solute supersaturation levels in stone formers. To increase urine volume, fluid intake greater than average may not by itself overcome the effects of living or working in a high ambient temperature, physical activity or chronic diarrhoea [46, 47]. Thus, the patient should achieve a goal of urine output rather than a set fluid intake [42] and in this regard, the 24-h urine volume is a very useful measure.

Urine pH, citrate, calcium and oxalate are dependent on factors in addition to diet and, therefore, cannot be used as reliable indicators of dietary intake. Citrate and pH depend on the balance of alkali intake and intake of dietary acid precursors, as well as loss of alkali from the gastrointestinal tract and physiologic abnormalities such as metabolic syndrome. Only a fraction of dietary calcium is absorbed, and that fraction varies from person to person. Urine calcium may also derive from bone mineral; furthermore, it is influenced by sodium and protein intake [48]. All in all, this makes calcium intake difficult to assess from urinary excretion alone. Oxalate has variable intestinal absorption and 50% or more of urine oxalate derives from endogenous oxalate production. Moreover, the calcium content of the diet itself influences the intestinal absorption of oxalate, probably because the formation of insoluble calcium oxalate in the food mixture reduces the oxalate available for intestinal absorption [49–51]. Indeed, a recent paper has demonstrated that high calcium ingested simultaneously with food intake reduces oxaluria and significantly also urinary CaOx supersaturation in idiopathic calcium oxalate stone formers [52].

### Consensus on Q4

The Consensus Group recognizes that although urine chemistry has limitations as an indicator of dietary intake, it nevertheless provides important information about diet that cannot be easily obtained by other means, and which can be valuable for managing treatment of the individual stone former.

### Q5. How should urine collections be made—(a) in a container which does not contain a preservative of any kind, (b) in a container to which preservative has been added before collection, (c) in a container to which preservative is added after collection or (d) some other method of collection?

The addition of stabilizers and the conditions under which urine is collected and stored represent important aspects to consider in the determination of 24-h urinary constituents. In most situations, a 24-h urine specimen cannot be analysed immediately, and so an additive that will inhibit the growth of microorganisms in the specimen is necessary [53, 54]. One approach for this has been to add thymol as a preservative to the container before collection (at about 1 g thymol per litre capacity of the container [54], and this method appears to preserve all important urine constituents to be measured [55, 56]. Other preservatives (such as chlorhexidine) have also been used [57]. Another approach has been to add acid (typically boric acid [1]) to the container before collection, as is commonly done for urine collected for microbiological analysis [58]. However, if acid is added as a preservative, the pH of the urine may need to be measured in an additional independent specimen, and it should be recognized that the pH of a spot urine is unlikely to correspond with that of a 24-h specimen [28].

### Consensus on Q5

The Consensus Group agreed that a 24-h urine specimen should be collected in the presence of a preservative, and that use of a non-acidic preservative simplifies the measurement of all parameters needed for supersaturation calculations.

## Q6. How should collected urine be handled before aliquoting for different analyses?

When a 24-h urine specimen is received (that is preserved with a non-acidic agent), its pH should be first measured using a pH meter (NOT by dipstick), and then the specimen thoroughly mixed. Some reports indicate that urine can then be aliquoted for various analyses without any further processing [59, 60]. However, studies specifically with urine from stone formers have shown that acidification of an aliquot is important for proper dissolution of crystals for the measurement of calcium and oxalate [61–63], and that alkalinisation of another aliquot is important for dissolution of uric acid crystals when they are present [63, 64].

### Consensus on Q6

The Consensus Group recommends that when a urine specimen arrives in the laboratory, its pH should be measured immediately using a pH meter. After vigorous agitation, one aliquot should be acidified (to pH of < 2) and another alkalinised (to 6.5 or higher) for dissolution of crystals that may be present in the specimen.

### Q7. What analytes should be measured in urine and why? Should we distinguish between samples collected for the routine screening of patients and samples collected for specific studies?

The constituents to be measured in a 24-h urine specimen are defined by the needs of diagnosing disorders and for planning treatment with recurrent stone formers. The volume of the 24-h urine specimen is an important measurement, and counselling stone formers to increase urine volume is a common and effective form of treatment [65]. For analytes, pH is essential for the calculation of the relative supersaturation of urine with respect to all potential stone constituents, but particularly calcium phosphate and uric acid. It can also be an indicator of stone type when its value lies within certain well-defined ranges. Other urinary constituents which the Consensus Group recommends for routine screening are calcium, oxalate, citrate and uric acid as they are potential indicators of hypercalciuria, mild and hereditary hyperoxaluria, hypocitraturia and hyperuricosuria, respectively, all of which are well established risk factors for stone formation. Urinary sodium should be measured to assess dietary salt intake, as described in question 4 above, and for its potential link with hypercalciuria. Additional constituents need to be measured particularly for accurate calculation of supersaturation values [66]. These include potassium, magnesium, phosphate, chloride, sulphate and ammonium. The latter two constituents are also recommended as important markers of dietary composition. Creatinine is useful for assessing the completeness of a given patient's 24-h collection, particularly if the patient is being followed up over a period of time. Urea is useful for assessing total protein intake, as mentioned earlier in this report. Finally, it is recommended that cystine screening be performed at least once in each patient to rule out this genetic cause of stones, an easy diagnosis which is too often neglected [67].

### Consensus on Q7

The consensus is that relatively few urinary constituents are needed for the routine clinical workup of patients (volume, pH, calcium, sodium, oxalate, citrate, uric acid, urea, creatinine) to provide initial insights about possible pathogenic conditions and to enable more complete dietary recommendations, but that several more are required to enhance the accuracy of supersaturation calculations (potassium, magnesium, phosphate, chloride, sulphate and ammonium).

## Q8. What inhibitors and/or promoters of crystallization should be measured in urine—if any?

The only substances with relative inhibitory capacity that are at present routinely determined in urine are citrate and magnesium, and it is likely that the actual effect of these two ions is primarily through reduction of supersaturation of calcium oxalate and calcium phosphate, rather than through direct effects on crystallization [68]. Citrate shows some ability to inhibit the crystallization of calcium salts, but probably its most important role is related to its ability to form soluble complexes with calcium, which results in a decrease in the supersaturation levels of insoluble calcium salts [69]. Magnesium, which can also exert a certain capacity to inhibit the formation of calcium salts, plays its most important role in the formation of a soluble species with the oxalate ion, which leads to a decrease in the level of supersaturation of urine with respect to calcium oxalate and calcium phosphate [68].

It seems certain that macromolecules in urine play an important role in the in vitro inhibition and promotion of crystallization [70], but there is little understanding of which, if any, of these molecules are useful for planning treatment in stone formers. Both osteopontin and Tamm-Horsfall glycoprotein have been shown in laboratory settings to inhibit crystallization [71], and excretion of both have been found to be lower in stone-formers [72, 73]. However, data on how such measurements might be used in governing the treatment of stone formers are lacking.

### Consensus on Q8

The Group agreed that more research is needed into the action of inhibitors and promoters of crystallization in urine, and how their concentrations can be clinically managed, before measurement of any of these becomes a regular part of the screening and treatment of stone formers.

## Q9. What are the best methods for analysing urine for the analytes specified in Q7?

Most of the constituents to be analysed in urine can be measured using the methods already established in clinical laboratories but each laboratory should confirm that the preservation method used (e.g., thymol, boric acid, etc.) does not interfere with any of the assays performed on the sample. Because assays vary in subtle details between manufacturers, this issue must be addressed by each individual laboratory.

Standard methods are in place in any clinical laboratory for the measurement of volume, pH (by meter, and not by test strip or dipstick), calcium, potassium, sodium, magnesium, phosphate, creatinine, urea, chloride. For oxalate, either enzymatic or chromatographic methods have been shown to work well with urine, but oxalate analysis can be especially sensitive to interference by other compounds, so laboratories must vet their procedures with care [61, 74]. It should be noted that to ensure complete dissolution of crystals of calcium oxalate in urine, it is essential to acidify the aliquot to < pH 2, particularly if the urine has both a raised oxalate AND calcium content [57]. However, this may raise a problem with the subsequent analysis of oxalate in the acidified sample if one of the enzymatic methods is used for measuring oxalate. The enzymes concerned work optimally at pH levels approximately between 3.5 and 5.5. This requires addition of significant amounts of alkali to be added to neutralize the added acid with a resultant marked increase in the ionic strength of the mixture. In turn, this may inhibit the full functioning of the enzyme and lead to an underestimate of the oxalate content of the urine [75]. Measurement of oxalate is best carried out by HPLC [76] or by ion-chromatography [77], but attention should be paid to the possibility of high pH in the eluent leading to conversion of ascorbic acid to oxalate [78]. Citrate can be measured by enzymatic or chromatographic methods [79]. Uric acid is commonly measured in automated systems using an enzymatic method [80].

Both ammonium and sulphate (or S) determinations are uncommon. Urinary ammonium can be measured by enzymatic methods using automated systems [81, 82], and the potential exists to use electronic methods [83, 84]. For sulphate, either precipitation with barium chloride or chromatographic methods is successful [85].

For cystine determination, the colorimetric nitroprusside technique works well [86], as do some chromatographic methods [87]. However, analysts should note that assays may not distinguish cystine from soluble thiol drug-cysteine complexes. This would be important in patients who are taking tiopronin or D-penicillamine, which form thio-cysteine bonds to increase cysteine solubility in urine. However, thiol-cysteine bonds can be broken during sample preparation, releasing cysteine which recombines with itself to form cystine. The result is inaccurate measurement in patients taking thiol drugs and inability to judge drug efficacy. An approach in managing cystine patients and judging the effectiveness of medication has been to measure the cystine capacity of the urine, a separate analysis that uses a solid phase method [88, 89]. Newer technology may also allow improvements in treatment of cystine stones [90].

### Consensus on Q9

The Consensus Group concluded that analysis of most analytes needed for proper assessment of 24-h urine collections is standard in most countries. The group agreed that more research is needed into the development of an easy and fast method for the separate detection in urine of soluble and insoluble cystine to monitor treatment in patients with cystinuria.

## Q10. Are there any specific tests available that patients can usefully perform at home to assess their risk of forming stones?

Increasing urine volume is a common form of treatment for stone patients, and a simple qualitative monitoring of urine colour [91] or urine conductivity [92] have been reported as being useful to patients who are seeking to maintain an appropriate intake of fluids. For some kinds of stones, the value of the urine pH is an important indicator of treatment success [93] and tracking this through each day using a dipstick has been recommended for cystinuric patients [1]. New devices that can provide more accurate measurement of urine pH by the patient at home are becoming commercially available [94, 95]. Potentially such devices could also provide other measures of urine composition.

If such measures are to be employed, it is important to counsel the patient on how to carry out the analyses at home with proper technique, and for many patients, overcoming their reluctance to do anything with their own urine can be a challenge. However, research is needed in this area, as there is little known about how successful home monitoring of urine can be in reducing the rate of stone recurrence.

### Consensus on Q10

The Consensus Group accepts that there are tests that patients can perform on their own urine at home that may be of (minor) utility, but proper counselling of patients is essential. However, it is recognized that these tests will merely serve as an indicator of progress to the patients themselves rather than being of clinical value to the prescribing physician. Home monitoring of the urine pH is an important measure to self-adjust bicarbonate/citrate treatment in cystinuric patients.

## Q11. Is there is value in calculating relative supersaturation in 24-h urines?

Urine is said to be supersaturated with respect to a given salt or acid when its activity product (a chemical measure of how much of a particular salt or acid is contained in a solution) exceeds its solubility product at which point the urine is said to be completely saturated with the salt or acid concerned. When urine is at the solubility product of that salt or acid it is defined as having a relative supersaturation value of 1. In the case of calcium oxalate, urine from normal adults may have relative supersaturation levels between 4 and 10 [96] but in the urine of untreated stone-formers this may reach values of 20 or more [22]. For comparison, the relative supersaturation of normal urine with respect to calcium phosphate is in the range 0.4–2.3 [96] but in untreated stone-formers may reach values of 10 or more [22]. For uric acid, the relative supersaturation of normal urine is in the range 0.4–1.9 [96] but in untreated stone-formers may reach values up to 3.5 [22].

In general, an increase in the relative supersaturation level of any of these salts or uric acid is consistent with the mineral deposited in stones [97], and so it makes sense to institute treatment modalities that are designed to decrease urine supersaturation levels in stone-formers. Indeed, reduction in the relative supersaturation of urine with respect to calcium oxalate correlates with a lower risk for calcium oxalate stone recurrence [18].

Measurement of urinary supersaturation of stone-forming salts and uric acid has been regarded as the gold standard for determining the risk of stone formation for over 50 years. Although supersaturation can be determined empirically, sophisticated computer programs have been widely used to calculate this important urinary property. Examples include SUPERSAT [29], EQUIL2 [98], JESS [99], and LithoRisk [100]. These programs generate similar but non-identical values for supersaturation depending on the number and type of urinary constituents that are used in the various calculations. Lowering mineral supersaturation values by means of dietary adjustment or relevant medication has been and continues to be the goal of most treatment regimens as it provides a numerical indication of potential efficacy in reducing the likelihood of stone recurrence. Several members of the Consensus Group stated that they seek to reduce calcium oxalate urine supersaturation levels in the urines of calcium oxalate stone formers to half of their initial value, but consensus was not agreed on a definitive target value for this objective.

### Consensus on Q11

The Group agreed that calculated relative supersaturation values provide a better indication of the propensity for crystals to form than will the measure of any single analyte in urine, and that these can be useful in directing treatment of stone formers and in assessing the efficacy of treatment.

## Q12. Is there any value in using indices of stone risk that seek to predict stone recurrence?

As discussed above, values for urine supersaturation can be useful in identifying urine properties to be targeted for therapy (low volume, high calcium, etc.) and for assessing the success of therapy (indicated by a reduction in urine supersaturation). But other efforts have been made to use urine analyses (or other data) to provide prediction of the probability of stone recurrence in a given patient, in part to reduce the number of analyses necessary for the calculation of relative supersaturation using sophisticated computer programs. These indices also can have the value of integrating data in ways that avoid any controversy about cut-off values for what would be ‘normal’ in analysis results [16].

The two Tiselius Activity Product (AP) Indices for calculating the risk of forming calcium oxalate and calcium phosphate stones are both relatively simple quotients involving urine constituents that together require only six measurements in urine: pH, calcium, oxalate, citrate, magnesium, and volume [21]. High values of these Indices have been shown to correlate with stone recurrence [101]. The Robertson set of risk indices (*P*_SF_) is another alternative for assessing stone risk [102]. Altogether these indices require seven urinary measurements for assessing the biochemical risk of forming calcium oxalate and calcium phosphate stones, but they also calculate the risk of forming uric acid stones. These indices correlate with actual stone recurrence [66]. The BONN-Risk Index relies on a laboratory estimation of the resistance of a urine sample to support the crystallization of calcium oxalate [103]. This is a specialized test procedure that includes an implicit assessment of the levels of promoters and inhibitors of crystallization in the urine and has been claimed to discriminate between a population that was prone to form stones and one that was not [104].

None of these measures has become widely adopted by physicians treating stone patients, and none has been assessed in trials to evaluate how well they can predict stone recurrence in a given patient. Yet, each shows promise to be able to identify patients who are at risk of stone recurrence.

### Consensus on Q12

The Consensus Group recognizes that several urine-based risk indices have been proposed, but that as yet none has the support of clinical trials to establish any of them as a standard predictor of stone recurrence in patients. The Group recommends that research in this area would be of great value to the stone treatment community.

### Q13. How should the results of screening tests be reported to the urologist or nephrologist treating the patient and how should the results be conveyed to the patient in a patient-friendly format?

The results of 24-h urine measurements can appear as a long list of numbers that will be difficult to interpret for the inexperienced physician, and incomprehensible to the patient. Thus, a simplified graphical format for presenting these data can go a long way toward making them broadly useful within the stone treatment community. It may also be a valuable means to convey in a simple visual way the results of urine analysis to the patients concerned.

**Fig. 1:**
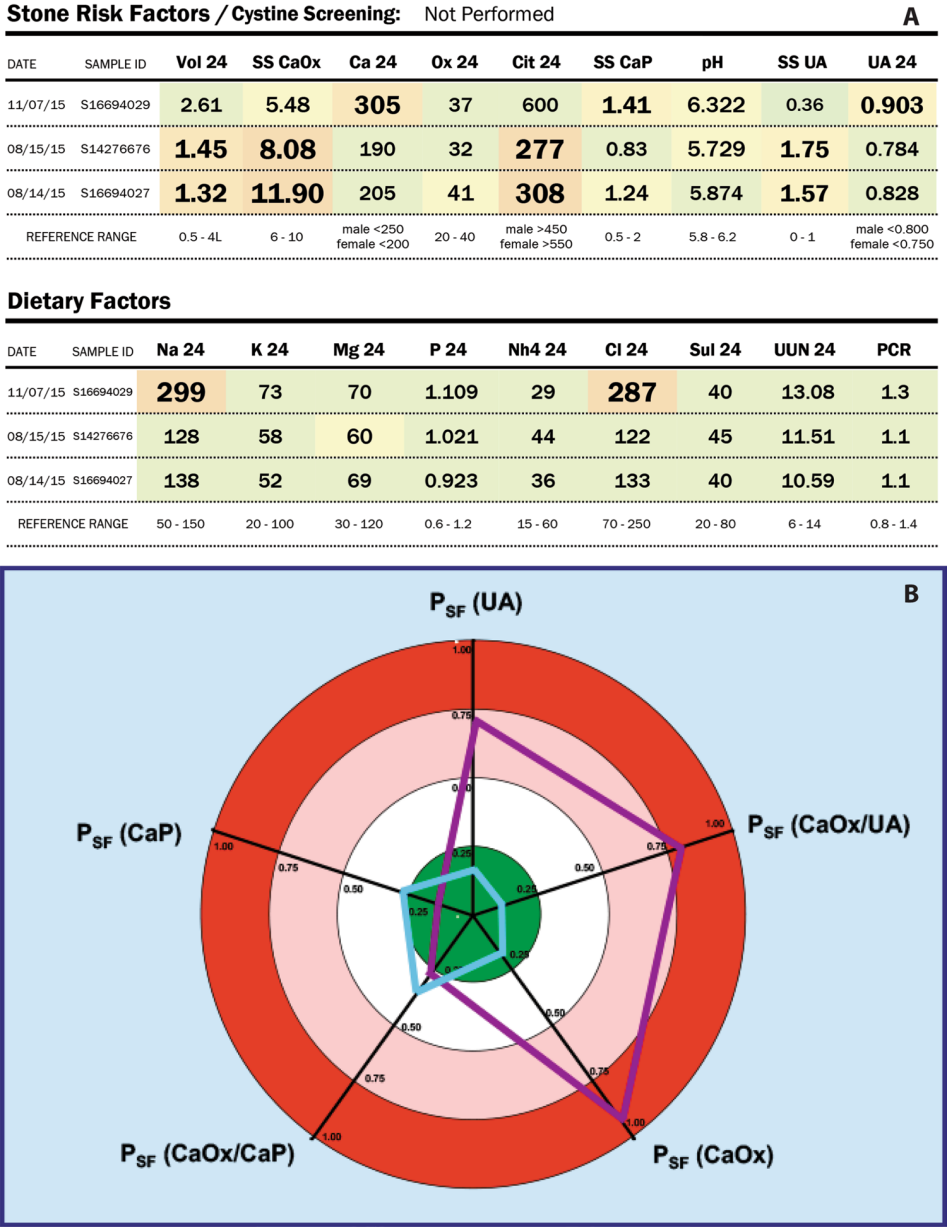
Examples of urine analysis reports that include graphical features to aid in interpretation. **a** Part of the LithoLink report (Laboratory Corporation of America, Burlington, NC, USA) for 24-h urine results. Note that measures that are out of the normal range are highlighted both by a change in background colour and an increase in the size and boldness of the typeface. The history of the patient is also shown, with the most recent results on the top line. Note that in this case, the patient has significantly increased the urine volume, and thereby reduced the supersaturation value for calcium oxalate (SS CaOx). However, this was done along with a dramatic increase in dietary salt (Na 24), which likely led to the increase in urine calcium (Ca 24), which was part of what drove an increase in the supersaturation value for calcium phosphate (SS CaP). **b** Part of the graphical report for urine results from the LITHOSCREEN system for assessing stone patients [29]. Note that targeted values (least likely to lead to a stone recurrence) are at the centre of the diagram, in the green bull’s eye. PSF indicates the Robertson biochemical risk of forming stones as described above under Q12. The initial untreated PSF values for the patient are shown in the purple lines and shape, and the values after treatment are shown in light blue. Before treatment, this patient was at risk of forming both uric acid and calcium oxalate stones or a mixture of the two. Following suitable dietary treatment, the PSF values of the patient all fell into the green bull’s eye. Similar target diagrams are also available for both 24-h urine and dietary composition [29]

Two examples of how reports might be presented are shown in Fig. 1. The upper panel shows part of a 24-h urine analysis report in which the colour of each box in the table and the size of typeface draw the eye to values that increase the chances of stone formation. If one reads the report from bottom-to-top, one sees the progress of the patient over time. The lower panel shows another approach, in which risk indices calculated from urine data are displayed in the form of a coloured Target Diagram, where values distant from the centre (i.e., in the red zone of the target) indicate a higher risk for stone recurrence and the values that the patient should be targeting to be safe from stone recurrence are shown in the central, green bull’s-eye.

These are just two examples, and within our Consensus Group, a number of laboratories presently utilize a variety of such graphical approaches to enhance readability by the physician and to provide effective ways to convey results to a patient. Note that both examples in Fig. 1 allow the physician to show the patient what changes have occurred with alterations in diet or medication. This kind of graphical information is likely to improve patient motivation and help advance the physician–patient relationship over the course of monitoring stone disease within a given individual.

### Consensus on Q13

The Group strongly recommends that results of 24-h urine analyses should be communicated in a manner that enhances understanding in both physician and patient, and that physicians should consider using any of the graphical methods which are available.

## Q14. Is there value in studying crystalluria?

The presence of crystals in urine is *prima facie* evidence that the balance between solute supersaturation and promoters on one hand and inhibitors of crystallization on the other has tipped toward precipitation. As such, crystalluria can provide evidence of the propensity of the urine to form stones [105]. However, it is recognized that this is not a conclusive diagnostic as some non-stone formers form crystals in their urine [106, 107], although these crystals, at least for calcium oxalate, are reported to be smaller and less aggregated than those found in the fresh urine samples from recurrent stone-formers [108].

Studies of repeated urine specimens for crystalluria have shown that this kind of crystalluria (large crystals, aggregated crystals) to be strongly correlated with stone recurrence [22, 108]. Furthermore, a more recent study of 188 patients over 3 years with multiple urine specimens showed that having 50% or more urine samples with crystals was predictive of stone recurrence with a sensitivity of 88% and a specificity of 84% [14]. This study is very suggestive that persistent crystalluria accurately reflects a propensity for stone formation. Similar results were seen in cystine patients, in which the volume of cystine crystals was also seen as highly predictive of those patients who would form a new cystine stone [109].

Additionally, examination of urine crystals can reveal rarer types of stone disease. For example, the crystals of cystine and 2,8-dihydroxyadenine in urine are very distinctive, easy to identify under the microscope, and pathognomonic of these two genetic diseases [105, 110].

Unfortunately, obtaining the proper urine specimens to determine crystalluria requires patients to submit specimens early in the morning, and for the specimens to be examined promptly (see comments in Q15 below). In addition, laboratory personnel must be skilled in assessing urinary crystals by microscopic examination. All these issues currently make assessment of crystalluria rare.

### Consensus on Q14

Crystalluria provides a natural indication of the propensity for stone formation, and thus would be valuable in assessing the probability of stone recurrence and in indicating if treatment is efficacious. Availability of crystalluria determination in more laboratories would likely benefit patients who are motivated to reduce their risk of stones. However, the Consensus Group recognizes that research in this area is needed for standardization of results and for the development of easy-to-perform evaluation methods (and possibly automatization) so that the use of crystalluria in diagnosis and treatment can become a routine part of care for stone formers.

## Q15. How should crystalluria best be assessed?

In most laboratories presently assessing crystalluria, it is thought that examination of the first morning urine yields the most crystals, reflecting the propensity of minerals to precipitate in the urine when it is most concentrated. However, unless the patient lives in close proximity to the analysis laboratory, this is unlikely to be practical. Thus, typical analyses are done using the second morning urine, which is collected midstream, while on premises at the laboratory, and immediately submitted for analysis [111, 112]. Urine is kept at room temperature and processed quickly (ideally within 20 min, but certainly within 2 h). Urine pH should be measured by meter. An aliquot of the urine is centrifuged to concentrate the specimen 20-fold, and then 50 µl pipetted onto a slide and topped with a coverslip.

These laboratories generally record crystal counts as number per high-power field, and crystal types are identified using a combination of bright-field and polarization microscopy [105, 110]. With this method, crystalluria can be expressed as mild (1–5 crystals per high power field), moderate (6–10), severe (11–20), and very severe (> 20). Alternatively, it is possible to quantify crystal content of urine using machine measures [108, 113], an approach that may be more likely to be automated in a way that assessment of crystalluria could be made more widely available. As mentioned above under Q14, the development of automated evaluation methods for assessing crystalluria would be useful, as would be the standardization of criteria for measurement and reporting of urinary crystals. Research in these areas is needed.

### Consensus on Q15

For present practice, the Consensus Group recommends that crystalluria should be determined by microscopy in morning urines by a skilled observer. The Group also recognizes that research into new methods, standardization of reporting criteria, and further research into how to apply test results for patient diagnosis and treatment will all be important for making crystalluria determination more widespread in the treatment of stone diseases.

## Q16. How should kidney stones best be analysed?

All published guidelines on treatment of stone formers include stone analysis as a first step in classifying the patient. Indeed, assessment of the results from the analysis of urine is impossible to do properly without knowing the kind of stone produced.

Stone analysis begins with examination of the stone under a stereomicroscope to assess which part (or parts) of the stone should be taken for molecular analysis. Most stones contain more than one mineral [114], and identification of visually distinct parts of the stone for dissection is important for correct analysis of minor constituents [115]. Stone portions taken should then be analysed by a molecular method, typically by either infrared spectroscopy or X-ray diffraction to identify mineral types [116]. Whatever method is used, it is important that it be able to distinguish, for example, brushite from other forms of calcium phosphate, and calcium oxalate dihydrate from monohydrate.

It is relatively simple to identify the morphological types of stones. This can significantly support clinically relevant information [117, 118]. This is done during the initial examination by stereomicroscope and typically also includes visual examination of the interior of the stone. It is easy, for example, to distinguish a calcium oxalate stone that formed initially as the dihydrate, which is indicative of the presence of hypercalciuria [119], even when its composition will have transformed over time to the monohydrate form of the mineral.

As discussed for the measurement of crystalluria, above, it is recognized that the development of methods of stone analysis that do not require a skilled observer would be valuable in enabling analysis of stone morphology and composition to be carried out more widely across the globe.

### Consensus on Q16

The Consensus Group strongly recommends that stone analysis be performed if possible as part of the workup of stone patients. The analysis should be conducted using infrared spectroscopy or X-ray powder diffraction. Visual identification of stone morphology is also valuable.

## Q17. Is there any value in analysing stone fragments?

Analysis of whole stones yields information on morphology and composition, but also allows discovery of the manner in which the stone was retained during early growth. Specifically, the identification of stones that have grown on Randall’s plaque is easy to perform [120]. However, many methods of stone removal (e.g., shock wave lithotripsy, laser lithotripsy) result in the fragmentation of stones.

Though information of how a stone might have been formed (such as the presence of Randall’s plaque) is lost when stones are fragmented, analysis of the fragments still allows for the mineral composition to be determined. Sometimes the general morphological class of the stone can also still be identified [117]. A general principle for stone analysis is that the more complete the specimen, the better will be the quality of the analysis [121], so submission of collections of fragments for analysis will generally be better than sending just a few.

### Consensus on Q17

The Consensus Group recommends the analysis of fragments, because knowledge of a stone’s mineral composition (albeit only partial) is an integral part of interpreting 24-h urine results and in planning treatment.

## Conclusions

The Consensus Group concludes that analyses of urine and stones should be routine in the diagnosis and treatment of urinary stone diseases. At present, the 24-h urine is the most useful type of urine collection, and methods for analysis and standards for interpretation are widely available. Patient education is also important for obtaining a proper urine sample. Graphical methods for reporting urine analysis results can be helpful both for the physician and for educating the patient as to proper dietary changes that could be beneficial. Proper analysis of stones is also essential for diagnosis and management of patients. The Consensus Group also agrees that research has shown that evaluation of urinary crystals could be very valuable, but the Group also recognizes that existing methods for assessment of crystalluria do not allow this to be part of stone treatment in many places.

## Electronic supplementary material

Below is the link to the electronic supplementary material.

Supplementary file1 (DOCX 20 kb)
