# Supplementary material for: Urine and stone analysis for the investigation of the renal stone former: a consensus conference
Source: Urolithiasis. 2020 Oct 13;49(1):1–16. doi: 10.1007/s00240-020-01217-3 (PMC7867533; doi:10.1007/s00240-020-01217-3)
Supplement: Supplementary file 1 — Supplementary file1 (DOCX 20 kb) [file 240_2020_1217_MOESM1_ESM.docx]

Table 1. Published guidelines regarding urine analysis for stone formers.

|  | **AUA** | **CUA** | **EAU** | **UAA** |  | Note | |
| --- | --- | --- | --- | --- | --- | --- | --- |
| CHILDREN  < 18 YRS |  | YES | YES | YES | ******* |  |  |
| FAMILY HISTORY | YES |  | YES | YES | ****** |  |  |
| URIC ACID URATE |  | YES | YES | YES | ***** | - Only urinary uric acid for pure UA  - Mixed CaOx/UA should be screened as calcium stone formers |  |
| CYSTINE |  | YES | YES | YES | ***** |  |  |
| BRUSHITE |  | YES | YES | YES | ******* |  |  |
| SOLITARY KIDNEY | YES | YES | YES | YES | ***** |  |  |
| ANATOMICAL ANOMALIES |  |  | YES | YES | ***** |  |  |
| NEUROGENIC BLADDER |  |  | YES | YES | ***** |  |  |
| MSK - POLYCYSTIC |  |  | YES | YES | ******* |  |  |
| NEPHROCALCINOSIS |  |  | YES | YES | ******* |  |  |
| GOUT | YES | YES |  |  | ***** |  |  |
| SARCOIDOSIS |  |  | YES | YES | ***** |  |  |
| INTESTINAL MALABSORPTION | YES | YES | YES | YES | ****** | Only urinary oxalate and citrate |  |
| HPT | YES | YES | YES | YES | ******* |  |  |
| RTA TYPE 1 | YES | YES | YES | YES | ******* |  |  |
| PRIMARY HYPEROXALURIA |  |  | YES | YES | ******* |  |  |
| OBESITY | YES |  |  |  | * |  |  |
| DIABETES TYPE 2 | YES |  |  |  | * |  |  |
| METABOLIC SYNDROME |  |  | YES | YES | * |  |  |
| CYSTIC FIBROSIS |  |  | YES | YES | *** |  |  |
| 2,8 DHA  XANTHINE  LESCH-NYHAN S. |  |  | YES | YES | * |  |  |
| LEAD EXPOSURE |  |  | YES | YES | * |  |  |
| # COLLECTIONS | 1-2 | 2 | 2 | 2 | *** |  |  |
| ●AUA, American Urological association; CUA, Canadian Urological Association; EAU: European Association of Urology; EAU, European Association for Urology; UAA, Urological Association of Asia  ●Level of support from present consensus: * low; ** medium; *** high  ●Blue: recommended by all 4 organizations; Orange: recommended by 3 organizations | | | | | | |  |
